# Infantile Pertussis Rediscovered in China

**DOI:** 10.3201/eid0808.010442

**Published:** 2002-08

**Authors:** Jingmin Wang, Yonghong Yang, Jie Li, Jussi Mertsola, Heikki Arvilommi, Lin Yuan, Xuzhuang Shen, Qiushui He

**Affiliations:** * Beijing Children’s Hospital, Capital University of Medical Sciences, Beijing, China; † Turku University Hospital, Turku, Finland; ‡ National Public Health Institute, Turku, Finland

**Keywords:** pertussis, immunization, bacterial culture

## Abstract

Immunization against pertussis was introduced in China in the 1960s. Since the 1970s, no culture-confirmed pertussis cases have been reported in the country. We report six infants with culture-confirmed pertussis, who were initially diagnosed as having other respiratory diseases, at Beijing Children’s Hospital, Beijing.

Immunization against pertussis was begun in China in the 1960s. Three doses of whole cell pertussis vaccine combined with diphtheria and tetanus toxoids are given at 3, 4, and 5 months of age (1). Since 1982, a booster dose has been added, given at 18–24 months of age (1).

In the 1990s, >85% of children received at least three doses of vaccine, and the incidence of pertussis (based on clinical diagnosis) remained <1/100,000 population (1). In China, pertussis is a reportable infectious disease, diagnosed by physicians. Since the 1970s, no culture-confirmed pertussis cases have been reported in the country. However, during April–June 1997, a local outbreak of pertussis was reported in a rural village (population 1,387) in southwestern China (2). A total of 285 cases were diagnosed. The ages of these patients ranged from 6 months to 80 years; 44% were <7 years of age; and 23% were 15 years of age. No deaths were reported. The suggested cause for this outbreak was relatively low vaccination coverage in the village.

The diagnosis of pertussis, especially in patients with atypical symptoms, requires clinicians’ awareness and laboratory tools. To our knowledge, the current laboratory methods (e.g., culture, enzyme immunoassay serologic testing, and polymerase chain reaction assay [PCR]) are not being used to diagnose pertussis in China. In this study, we report six cases of culture-confirmed pertussis in infants seen at Beijing Children’s Hospital. All six patients were initially diagnosed as having other respiratory diseases.

## The Study

To determine how much bacterial culturing would aid the diagnosis of pertussis in China, a study was conducted in a 35-bed ward for respiratory diseases at Beijing Children’s Hospital from June 2000 to May 2001. This facility is the largest children’s hospital in China; it has 3,000–4,000 visits daily to its outpatient department. Nasopharyngeal (NP) swabs were taken from children who had been admitted to the hospital because their cough, with or without paroxysms, had persisted for >2 weeks and was worsening. A total of 55 children (age range 35 days to 13 years) were enrolled during the study period. In addition, NP swabs were obtained from two children (ages 10 weeks and 13 years) with paroxysmal cough who were seen at the outpatient department. Information about disease history, immunization status, and cough characteristics was obtained. Physical examination, chest X-ray, and blood tests were performed. At admission, all participants were diagnosed as having bronchitis, bronchopneumonia, or pneumonia.

After collection, NP swabs were immediately spread onto charcoal agar plates supplemented with cephalexin. Details of *Bordetella pertussis* culture have been described previously (3). In brief, after inoculation, the plates were incubated in a humid atmosphere at 35°C and inspected daily for 7 days to determine pertussis-like colony growth. Suspected colonies were Gram stained and tested by slide agglutination with antisera to *B. pertussis* and *B. parapertussis* (Murex Diagnostics, Dartford, England).

Serum immunoglobulin (Ig) G antibodies to purified pertussis toxin (PT) were tested by enzyme immunoassay, as described (3). Seropositivity was determined by comparing the antibody results in patient serum samples with those in 460 healthy Chinese persons. Results exceeding the mean of the controls by two standard deviations were considered to be seropositive.

## Conclusions

Six infants <4 months of age were culture positive for *B. pertussis* (Table). Five of these patients were in the study group of 55 hospitalized children; the other was one of two children seen at the outpatient department. Before they went to the hospital, all six infants had taken broad-spectrum antibiotics but not erythromycin. None had received any doses of pertussis vaccine.

**Table:** Clinical details of six infants with pertussis, China, June 2000–May 2001

Case	Age (weeks) and sex	Duration of cough at sampling (day)	Signs and symptoms	Leukocyte count X 10^9^/L (% lymphocytes)	Diagnosis at admission	Possible source of infection
1	5 (M)	15	Paroxysmal cough, cyanosis	15.3 (62.5)	Bronchopneumonia	Mother, father, grandmother^a^
2	9 (F)	15	Cough without paroxysms	19.8 (57.7)	Bronchopneumonia	Cousin
3	8 (M)	15	Paroxysmal cough	17.6 (63.5)	Bronchitis	Not known
4	11 (M)	15	Paroxysmal cough	26.7 (52.0)	Pneumonia	Mother, father
5	16 (M)	20	Paroxysmal cough, vomiting	14.0 (55.0)	Pneumonia	Mother, aunt, cousin^a^
6^b^	10 (M)	5	Paroxysmal cough	27.0 (69.9)	Bronchitis	Mother, grandmother

^a^Culture positive for *Bordella pertussis*. ^b^Patient was treated in the outpatient department but not hospitalized.

The immediate family members or other relatives of five infants (cases 1, 2, 4, 5, and 6) had concurrent and persistent cough (Table). NP swabs and serum samples were obtained from family members of cases 1, 5, and 6 (data not shown). Case-patient 1’s grandmother was culture positive for *B. pertussis*. She, as well as the patient’s mother and father, had been coughing for several weeks, and they all had IgG diagnostic antibodies to PT in their sera. Patient 5’s mother, aunt, and 10-year-old cousin had diagnostic serum IgG antibodies to PT, and another, 8-year-old cousin was culture positive. The grandmother of case-patient 6 had been coughing for 1 month and had diagnostic serum IgG antibodies to PT.

Because antigenic divergence, with respect to PT and pertactin (PRN), has been recently found between *B. pertussis* vaccine strains and circulating strains, the PRN and PT types of eight clinical strains isolated in this study and two vaccine strains were examined. The methods used for this genotyping were LightCycler (Roche Applied Science, Mannheim, Germany) real-time PCR and fluorescence resonance energy transfer hybridization probes (4,5). The two vaccine strains and seven clinical strains harbored *prn1*, and one clinical strain contained *prn2*. For PT types, the vaccine strains harbored *ptxS1B* or *ptxS1D,* and all clinical strains had *ptxS1A*.

To our knowledge, this is the first report of culture-confirmed pertussis cases from China during the last 30 years. Of the 55 patients hospitalized for persistent cough, 5 (9%) were culture positive for *B. pertussis*. These results indicate that pertussis is not uncommon in the Chinese population and still causes substantial illness in infants and young children, although the pertussis vaccination coverage for the first three doses is >85% (1).

Immunity from immunization wanes with time; thus, older children and adults become susceptible to pertussis (6–8). The role of older children and adults in transmitting *B. pertussis* to unvaccinated infants has been well documented. More than 80% of hospitalized infants are not the index cases in their families (9). Our results agree with these reports. The family members and other relatives of five infants with culture-confirmed pertussis started to cough first. Evidence of laboratory-confirmed pertussis was obtained from the family members and other relatives of three infants.

Although the most serious effects from pertussis occur in young infants (10,11), all six ill infants in our study recovered. Pierce et al. reported 13 critically ill infants with confirmed pertussis, all <3 months of age (11); 4 had leukocyte counts >100 X 10^9^/L; all these infants died. Nine had leukocyte counts <100 X 10^9^/L (11). In the six ill infants in our study, the highest leukocyte count was far lower, 37 X 10^9^/L. However, our six patients had taken antibiotics before consultation.

In this study, pertussis was not initially suspected in these ill infants. None had the characteristic whoop. Pertussis at this age group is likely to be atypical, and symptoms resemble those of other respiratory tract infection, apnea, or cyanosis. Consequently, the diagnosis of pertussis is not considered and the treatment is delayed (10). These six infants were initially diagnosed as having bronchitis, bronchopneumonia, or pneumonia. The fact that pertussis was not considered in the differential diagnosis may indicate that clinicians were not aware that *B. pertussis* was circulating in the community.

Early and correct diagnosis of pertussis is important for effective therapy and prevention of transmission of the disease. Culture of *B. pertussis* from NP samples usually takes 3–7 days. In comparison with culture, PCR is a more sensitive and specific method for diagnosing pertussis (3). Measurement of specific serum antibodies to *B. pertussis* antigens by enzyme immunoassay can also facilitate this diagnosis, and results are helpful for epidemiologic studies (3,7,12). The use of culture for the diagnosis of pertussis is now being considered in Beijing Children’s Hospital.

Our results suggest that a number of pertussis cases are likely being misdiagnosed in China and that the incidence of the diseases is underestimated.
